# Thyroid dysfunction in aging and cardiovascular-kidney-metabolic syndrome: metabolic regulation and clinical implications

**DOI:** 10.3389/fendo.2026.2000192

**Published:** 2026-10-22

**Authors:** Hui Li, Na Wang, Xianhui Ran, Zhiyuan Fan, Tianyi Zhao, Xiao Ma

**Affiliations:** 1 Health Checkup Center, China-Japan Friendship Hospital, Beijing, China; 2 State Key Laboratory of Respiratory Health and Multimorbidity, China-Japan Friendship Hospital, Beijing, China

**Keywords:** aging, cardiovascular-kidney-metabolic syndrome, metabolism regulation, thyroid dysfunction, thyroid hormones

## Abstract

Thyroid hormones are involved in multiple metabolic pathways in the body and are essential for maintaining organ functions across the lifespan. Aging-related thyroid dysfunction has a bidirectional relationship with the development of chronic diseases including metabolic syndrome, diabetes, and cardiovascular disease, and contributes to the progression of cardiovascular-kidney-metabolic (CKM) syndrome via complex pathophysiological mechanisms. Systematically elaborating on the characteristics of thyroid function related to aging and its regulatory role in metabolism and cardiovascular homeostasis, can provide new therapeutic strategies and theoretical basis for revealing the molecular network and metabolic process of the CKM syndrome. In this review, we have summarized the manifestations of thyroid hormone homeostasis in age-related changes, and the underlying potential mechanisms during aging and longevity processes. Moreover, we systematically elucidated the pivotal role of thyroid hormones in cardiovascular and renal homeostasis, discussed the interplay between thyroid dysfunction and metabolic risk factors in CKM syndrome, particularly issues related to obesity, type 2 diabetes, and dyslipidemia. Patients with thyroid dysfunction develop CKM syndrome through a positive feedback loop centered on metabolic disorders and organic dysfunction. Moreover, we pointed out that novel drugs targeting thyroid hormone receptors are expected to slow down the progression of CKM syndrome and reduce cardiovascular risks.

## 1 Introduction

Aging is a multi-dimensional biological process that leads to the progressive functional decline and adaptive impairment of tissues and organisms (1). The occurrence and development of various chronic diseases, such as cardiovascular diseases (CVD), diabetes, and chronic kidney disease (CKD), is believed to be associated with aging (2). With the intensification of population aging, the rising prevalence of age-related chronic diseases has increased multi-morbidity risks and posing challenges to global public health (3). CKM syndrome that issued by American Heart Association is defined as a systemic condition involving two or more interrelated factors: CVD, CKD, metabolic risk factors, type 2 diabetes (T2DM), and obesity (4). CKM syndrome markedly elevates risks for atherosclerotic cardiovascular disease, heart failure, and significantly impacting morbidity and mortality (5). Age-related metabolic alterations constitute one of the core features of CKM syndrome, driving research into its pathogenesis and potential interventions.

Currently, thyroid diseases still occupy a significant position in the global disease spectrum, including overt and subclinical thyroid dysfunction. Thyroid dysfunction impacts metabolism, cardiovascular, nervous and immune systems, elevating risks of aging-related chronic diseases (6). Emerging evidence links THs with aging, lifespan, and development and occurrence of aging-related disorders like CVD (7, 8). Investigating local TH regulation during aging process, and clarifying the role of thyroid metabolism in CKM syndrome, may yield novel therapeutic approaches for chronic diseases. This review systematically elaborates how age-related thyroid dysfunction impacts longevity and its association with CKM syndrome, while summarizing underlying mechanisms and research advances to provide potential intervention strategies for the elderly suffering from CKM patients.

## 2 Aging linked with thyroid status

### 2.1 Thyroid hormones in aging tissues

Under physiological conditions, THs are systematically regulated by hypothalamus–pituitary–thyroid axis that follows a typical negative feedback loop (9). Upon entering circulation, tissue-specific deiodinases convert thyronine (T4) into triiodothyronine (T3), which exerts main biological effects. Intracellular T3 interacts with TH receptors (THRα/β) in different tissues to regulate metabolism (9).

Studies have shown that elderly populations exhibit higher thyroid disease prevalence than the general population, with increasing incidence of hyperthyroidism and hypothyroidism in recent years (6). Among them, the prevalence of clinical hypothyroidism, subclinical hypothyroidism, and subclinical hyperthyroidism increases with age, while the prevalence of hyperthyroidism decreases. Subclinical hypothyroidism is the most common in the elderly, accounting for approximately 6% of the population aged 70 to 79, and rises to 10% among those over 80 years old (10). A retrospective study in Copenhagen indicated that subclinical hypothyroidism with thyroid stimulating hormone (TSH) ranging from 5 to 10 mIU/L was associated with a reduced risk of all-cause mortality in individuals over 65 years old (11). Furthermore, TSH levels ranging of 4.5-10.0 mIU/L may indicate better health status and lower mortality (4). This part will be discussed in the following section.

Population iodine status is a major factor influencing thyroid dysfunction in the elderly, especially hyperthyroidism. In iodine-deficient regions, the prevalence of hyperthyroidism increases with age, reaching as high as 15% in those over 70 years old (12). In iodine- sufficient areas, the prevalence of hyperthyroidism in the elderly is lower than that among general adults (13). Autoimmune Graves’ disease, the predominant cause in the elderly, often present with insidious-onset hyperthyroidism affecting cardiovascular health, frailty, and overall function. Multiple studies have shown that high free THs and low TSH levels reduce life-quality and increase mortality risk. For untreated elderly hyperthyroid patients, the CVD risk still significantly elevated even when hyperglycemia, dyslipidemia and hypertension are controlled (13).

In mice, the association between shortened lifespan and long-term elevated free THs reinforcing the role of THs in aging. Rodent studies show that the binding of T3 to THRβ induces tissue aging and DNA damage, accelerating premature aging (14). Different THR isoforms may mediate different tissue effects during the aging process. In pancreatic β-cells, THRα isoforms bind to the aging marker p16Ink4a, while THRβ1 bind to Mafa but not to p16Ink4a (15). These findings suggested the feasibility of promoting healthy aging or extending lifespan of mammals by regulating THs, but still with a long way to go.

### 2.2 Thyroid stimulating hormone and longevity

As mentioned before, scholars have long noted that aging is associated with pituitary-thyroid axis function changes and a higher prevalence of autoimmune and nodular thyroid diseases. For per decade in age beyond 40, the upper limit of the TSH reference range will increase by 0.3 mIU/L (16). Recent studies highlight relationship between THs and longevity. Cross-sectional studies from the National Health and Nutrition Examinations Survey III showed age-related TSH increases among the population without thyroid disorders (17, 18). Observational studies have found that centenarians (19) and nonagenarians (20) exhibit elevated TSH levels compared to controls. In addition, their descendants exhibited longevity genetic predisposition while their partners did not. Among 2290 high-functioning older adults (70–79 years) in the Health ABC Study, mild subclinical hypothyroidism (TSH 4.5-10.0 mIU/L) was associated with slightly better mobility and lower risk of functional decline. Despite limited evidence from large scale cohort study, researchers tend to believe that higher serum TSH levels and low free T3 levels are associated with longevity (20, 21).

Several mechanisms may explain age-related TSH alterations: including reduced TSH bioactivity itself, decreased tissue-specific availability of THs, diminished negative feedback sensitivity of pituitary-thyroid axis, and blunted thyroid response to TSH (**Figure 1**) (21). A longitudinal cohort by Bremner et al. revealed no evidence accompanying T4 changes, suggesting that changes in serum TSH are not the consequence of pituitary-thyroid axis feedback (21, 22). Current researches support no other potential mechanisms except for attenuated response of THs to TSH. On the one hand, TSH administration produces less THs in the longevous than in controls. On the other hand, the promoter-enhancer region of the thyroid stimulating hormone receptor (TSHR) gene exhibits unique single nucleotide polymorphisms in the longevity and their descendants, which is correlate with elevated TSH. This may reflect a more directly impact of TSH on the stability and homeostasis of FT3 with unknown specific mechanism.

**Figure 1:**
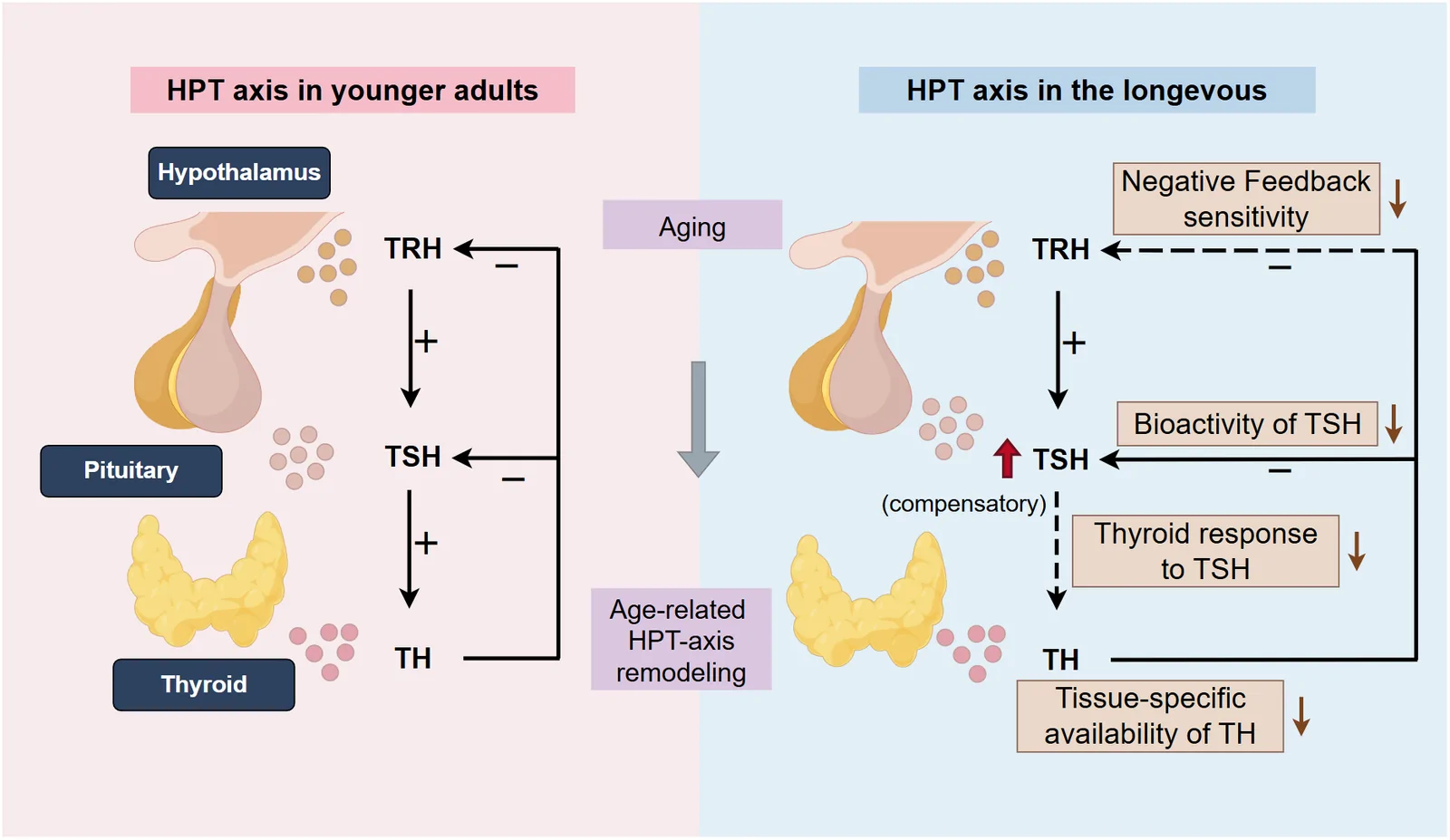
Hypothalamic-pituitary-thyroid axis may explain TSH alterations in the longevous. TRH, thyrotropin-releasing hormone; TSH, thyroid stimulating hormone; TH, thyroid hormone.

## 3 Cardiovascular diseases and thyroid dysfunction

### 3.1 Thyroid dysfunction and cardiovascular risk

Abnormal levels of circulating THs have been regarded as an independent risk factor for CVD. A meta-analysis (23) demonstrated the consistent correlation between FT4 levels and CVD as well as mortality. While subnormal TSH concentrations correlated with elevated CVD incidence and all-cause mortality. Among those over 70 years old, FT4 exceeding the 17.6 pmol/L in female and 18.4 pmol/L in male conferred over 5% and 10% 10-year risk increase, respectively (23).

Overt or subclinical hyperthyroidism induce tachyarrhythmias including sinus tachycardia, atrial fibrillation (AF), and atrial flutter, linked to high supraventricular ectopic activity. Approximately 10-15% of hyperthyroidism cases are accompanied by AF, that is appreciably higher than the incidence in the general population (24). The incidence of AF in hyperthyroidism demonstrates a marked age-dependent pattern, with a notably high prevalence in the elderly. More precisely, among those over 60 years old, 25% hyperthyroid patients suffer from AF, which is significantly higher than that those younger than 60 (25). The combination of AF in hyperthyroidism significantly increases the risk of embolism, stroke, heart failure, and cardiovascular mortality (24). In addition, among newly diagnosed AF patients, approximately 13% have hyperthyroidism. However, latest data casts doubt on evidence from previous studies by indicating that subclinical thyroid dysfunction and euthyroid individuals share similar cardiovascular risk profiles (26).

### 3.2 Aging-related chronic heart failure

#### 3.2.1 Hyperthyroidism and heart failure

Hyperthyroidism manifests as a hyperdynamic circulatory state with elevated cardiac output by 50%-300%, and is characterized by tachycardia, enhanced myocardial contractility, and decreased peripheral vascular resistance (12), as shown in **Figure 2**. Long-term severe hyperthyroidism, as detected by speckle tracking echocardiography, lead to decreased left ventricular ejection fraction (LVEF) and altered overall longitudinal strain, with approximately 6% progressing to left ventricular systolic dysfunction-induced heart failure (27). In elderly hyperthyroidism patients, exertional dyspnea and edema often represent initial manifestations that indicate congestive heart failure. In addition, for patients with symptomatic heart failure and an LVEF <35%, hyperthyroidism is an important independent prognostic factor and increases mortality risk by 85% (28).

**Figure 2:**
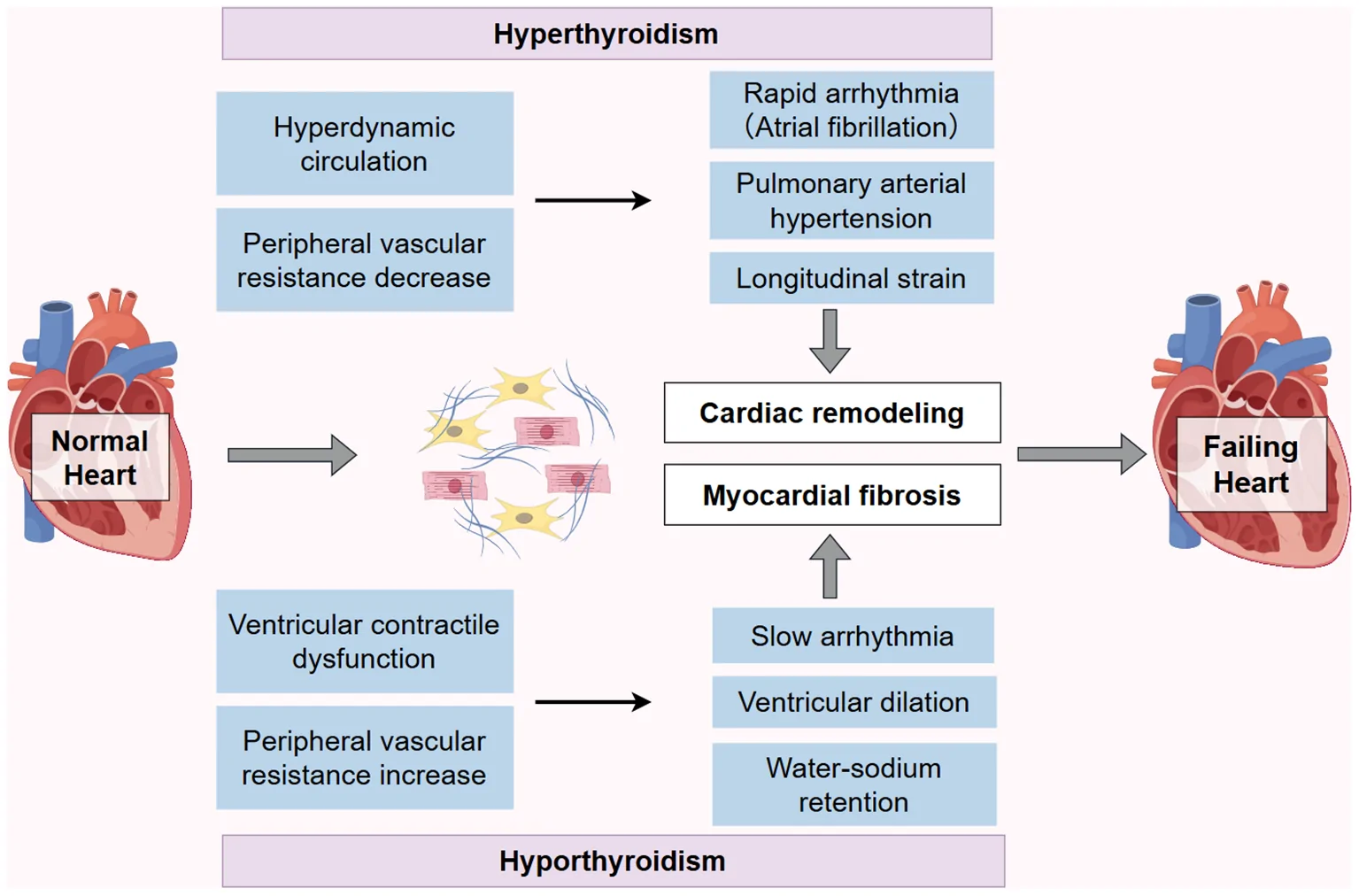
Underlying mechanisms of heart failure in hyperthyroidism and hypothyroidism.

Hyperthyroidism also promotes pulmonary arterial hypertension (PAH), observed in approximately 43% of patients (12). The mechanism may involve endothelial injury secondary to high cardiac output, upregulating pulmonary vasodilatory proteins and increasing pulmonary vascular resistance. In SuHx-induced pulmonary arterial hypertension rats, Al Husseini et al. found that thyroidectomy alleviated hypoxia-induced PAH, and treatment with propylthiouracil (PTU) reversed the formation of severe experimental PAH (29). Mechanistically, THs promote pulmonary arterial endothelial proliferation via α(v)β(3) integrin and fibroblast growth factor receptor, independent of pulmonary vascular resistance (29). The precise mechanisms linking hyperthyroidism and PAH progression warrant further investigation.

#### 3.2.2 Hypothyroidism and heart failure

Overt hypothyroidism is strongly associated with heart failure, primarily through reduced ventricular contractility and elevated peripheral vascular resistance, ultimately progressing to ventricular dilation, as shown in **Figure 2**. In PTU-induced hypothyroidism rats, Tang et al. demonstrated sustained ventricular dilation and contractile dysfunction (increased ventricular diameter-to-wall thickness ratio) in hypothyroidism (30). Research by Hajje et al. found concurrent upregulation of cardiac remodeling genes and fibrosis progression in hypothyroid rats (31). Rapid correction of thyroid function with L-thyroxine improved cardiac function in rats, while exacerbated myocardial inflammation and cellular infiltration (31). While a randomized, double-blind clinical trial also showed no significant improvement of left ventricular function with long-term oral T3 supplementation in hypothyroid heart failure patients (32).

The cardiovascular impact of subclinical hypothyroidism remains controversial. A prospective cohort study associated subclinical hypothyroidism with TSH>10 mIU/L with increased risk of heart failure (33). A meta-analysis from prospective studies confirmed elevated risk and mortality of coronary heart disease in subclinical hypothyroidism, particularly at TSH ≥10 mU/L (34). Notably, 33% of heart failure patients exhibit subclinical hypothyroidism, with higher prevalence in reduced ejection fraction cases. This apparent longevity paradox may reflect genetic variability (21). A comprehensive Genome Wide Association Study (GWAS) on thyroid function to date has identified 42 genetic loci associated with TSH levels and 21 loci linked to FT4 levels (35). Mendelian randomization analyses revealed that genetically predicted higher TSH levels within normal range, but not FT4 levels, were associated with a marginal elevation in cardiovascular risk (36).

## 4 Glucose and lipid metabolic homeostasis and thyroid dysfunction

### 4.1 Insulin resistance, diabetes and thyroid function

Diabetes mellitus (DM) and insulin resistance represent the most prevalent endocrine disorders, and exhibit a bidirectional pathophysiological relationship with thyroid dysfunction (37). Thyroid hormone directly regulates hepatic gluconeogenesis, and pancreatic β-cell function, and disorders in thyroid function reduces insulin sensitivity at these sites (38). Aberrant glucose metabolism in DM disrupts thyroid function, while thyroid dysfunction exacerbates insulin resistance in CKM patients (39).

A comprehensive retrospective cohort study demonstrated that compared to healthy controls, hyperthyroidism elevates the risk of both T1DM and T2DM, with adjusted hazard ratios of 1.66 and 1.71, respectively (39, 40). For patients with hypothyroidism, the relative risks of developing T1DM and T2DM are both 1.77 (40). However, in elderly patients with hyperthyroidism or hypothyroidism over 65 years old, the risk of T1DM and T2DM did not increase significantly (40). Interestingly, the study unexpectedly revealed that patients undergoing antithyroid drug therapy exhibited an increased diabetes risk (40). Similarly, those receiving thyroid hormone replacement therapy demonstrate higher diabetes risks (41). Mechanically, hypothyroidism promotes insulin resistance via glucose transporter 4 dysfunction, leptin resistance from autonomic dysfunction, and hyperleptinemia in diabetes. An early study also found that the A/G polymorphism of type 2 deiodinase leads to changes in the fT3 level in skeletal muscle, and is associated with insulin resistance in T2DM patients (42).

The NHANES studies demonstrate that the prevalence of thyroid dysfunction among American diabetic patients is significantly increased, especially among patients with anti-thyroid peroxidase antibody (TPOAb) positivity (41, 43). A systematic meta-analysis that included 38 studies revealed that the prevalence of thyroid dysfunction in patients with T2DM was 20.24% (44). Among them, the prevalence of subclinical hypothyroidism and hypothyroidism was 11.87% and 7.75% respectively; the prevalence of subclinical hyperthyroidism and hyperthyroidism was 2.49% and 2.51%, which was generally higher than that of the normal population (44).

Chronic hyperglycemia induces thyroid dysfunction through multiple interconnected pathophysiological mechanisms, including impaired sensitivity to circulating free THs, disrupted negative feedback mechanisms of HPT axis, damaged thyroid follicular epithelial cells leading to impaired hormone synthesis and release, and complex bidirectional interactions between disordered glucose metabolism and autoimmune processes (6, 45). Factors that increase the risk of thyroid dysfunction in T2DM patients include being female, having a glycated hemoglobin level of ≥7%, having a diabetes duration of more than 5 years, having a family history of thyroid dysfunction, having central obesity, smoking, having retinopathy and neuropathy, etc (39).

### 4.2 Dyslipidemia, obesity and thyroid dysfunction

#### 4.2.1 Hypothyroidism and dyslipidemia

THs potently regulate appetite and stimulate lipolysis. By enhancing lipase activity and adipose tissue catecholamine sensitivity, THs accelerate hydrolysis of triglycerides (TG) into free fatty acids (FFAs) and glycerol (46). They also promote mitochondrial β-oxidation of FFAs for energy production, increase lipoprotein lipase activity, enhancing clearance of very low-density lipoprotein (VLDL) and low-density lipoprotein (LDL), and promoting synthesis of high-density lipoprotein (HDL) (46). Additionally, THs modulate cholesterol metabolism by regulating synthesis, bile acid conversion, and biliary excretion (46). Thus, serum fT3 and fT4 levels critically determine lipid fluctuations, directly linking to lipid metabolism disorders in pathophysiology conditions.

Both overt and subclinical hypothyroidism markedly increase dyslipidemia including total cholesterol, LDL-cholesterol(LDL-C) and TG, and this is generally proportional to the degree of TSH elevation (47). Evidence from meta-analysis indicates that levothyroxine replacement therapy significantly ameliorates the atherogenic lipid profile in both overt and subclinical hypothyroidism, reduces LDL-C by approximately 41 mg/dL in overt cases (48). Given that dyslipidemia defines stage 2 of the CKM syndrome and drives early atherosclerosis, untreated thyroid dysfunction may accelerate vascular damage during this critical window (5). Clinical evaluation of thyroid status should be carried out in the elderly presenting with disproportional LDL-C levels to lipid-lowering therapy intensity (47).

#### 4.2.2 Hypothyroidism and obesity

Resting energy expenditure and thermogenesis are sensitive to thyroid hormone status, showing measurable declines even in the presence of minor reductions in circulating free thyroxine (38). However, the direct weight gain from this is usually limited and is largely attributable to non-adipose components. Specifically, the accumulation of hydrophilic glycosaminoglycans in the interstitium, coupled with impaired renal hemodynamic and fluid clearance, leads to myxedematous changes that mimic adiposity (49). Longitudinal assessments of body composition following levothyroxine initiation reveal that weight reduction is mainly due to the loss of water and lean tissue, while fat mass remains relatively stable. These findings indicate that severe obesity is seldom a direct consequence of hypothyroidism in isolation (49, 50).

Nevertheless, in the context of the elderly population and metabolic syndrome (MetS), the association between thyroid function and obesity has been widely investigated. A study based on Longitudinal Aging Study Amsterdam by Heima et al. established a connection between elevated circulating TSH levels and increased risk of developing MetS and obesity (51). Subclinical hypothyroidism was also independently associated with an increased risk of obesity, hypertension, high TG and low HDL-cholesterol levels (50). Another meta-analysis involved seven prospective studies indicated that both clinical and subclinical hypothyroidism risk significantly increased in individuals with obesity at baseline (52).

## 5 Thyroid dysfunction and CKM syndrome

CKM syndrome is an advancement concept of cardiorenal syndrome, emphasizing the bidirectional interaction between the cardiovascular system and the kidney, and revealing crucial role of systemic glycolipid metabolism disorders, inflammation, and insulin resistance in the development of cardiovascular diseases.

### 5.1 Pathophysiological mechanism of CKM and thyroid dysfunction

CKM is characterized by complex pathophysiological features involving intricate interactions between hemodynamic and neurohormonal mechanisms (4), such as excessive sympathetic overactivity, the renin-angiotensin-aldosterone system, and oxidative stress. Patients with thyroid dysfunction may develop CKM syndrome through a positive feedback loop centered on metabolic disorders and organic dysfunction (including CVD and CKD).

#### 5.1.1 The cardio-renal axis and CKM risk

The cardio-renal axis is a crucial physiological hub in CKM syndrome, and hemodynamics holds an important position within it (4). Imbalance of vascular tone, endothelial dysfunction, and hemodynamic instability jointly drive progression of overt cardiovascular and kidney disease (53). Thyroid hormones serve as critical modulators of this axis. Both hypothyroidism and hyperthyroid states impose significant hemodynamic stress on the heart and kidneys (54). Hypothyroidism induces a hypometabolic phenotype characterized by elevated systemic vascular resistance and impaired endothelium-dependent vasodilation, whereas hyperthyroidism has broadly opposite hemodynamic effects, and both disturb blood pressure regulation (24). Notably, even subtle elevations in TSH within the reference range were independently associated with higher systolic and diastolic pressures (55).

Concurrently, thyroid hormone deficiency compromises renal hemodynamics by reducing renal blood flow and glomerular filtration rate (GFR), while promoting tubular sodium and water retention (4). A meta-analysis of 15 studies involving approximately 5.1 million participants found that hypothyroidism was associated with an increased risk of CKD in both cross-sectional and cohort analyses (56), and thyroid dysfunction was still significantly prevalent in patients with end-stage renal disease and those receiving dialysis (57). Considering the high comorbidity risk of hypertension and CKD in the elderly, these findings position thyroid dysfunction as a critical upstream modifier within the CKM syndrome. By inducing endothelial injury, adverse hemodynamic remodeling, hypertension, and impaired renal filtration, thyroid abnormalities accelerate the pathological transition from metabolic risk to obvious cardiorenal disease pathological states.

#### 5.1.2 Converged dyslipidemia and CKM risk

Metabolic risk, renal impairment, and cardiovascular disease are interrelated in CKM syndrome, forming a mutually reinforcing cycle in which insulin resistance, dyslipidemia, and endothelial injury sustain (58). Dyslipidemia is the critical nexus where thyroid disease, diabetes, and CKM syndrome converge most clearly. The LDL-C and TG elevation caused by hypothyroidism, along with the characteristic insulin resistance, further promote the formation of the atherosclerotic lipid profile in the CKM (47). On the one hand, LDL-C, characterized by enhanced oxidizability and impaired clearance, drive endothelial inflammation and atherogenesis (46). On the other hand, insulin resistance leads to excessive production of small dense LDL particles from VLDL, which have prolonged circulation time and enhanced oxidizability. Small dense LDL is a more effective predictor of atherosclerotic CVD, and is closely related to fatty liver and CKD (59). Furthermore, thyroid dysfunction exacerbates the inherent chronic inflammation and endothelial activation in the pathogenesis of CKM (50). These mechanisms collectively indicate that thyroid dysfunction drives CKM progression through a continuous reinforcing loop, rather than a linear pathway, with dyslipidemia serving as a critical mechanistic hub.

### 5.2 Thyroid hormone sensitivity and CKM

In 2019, Martin et al. proposed the thyroid feedback quartile index (TFQI), converting FT4 and TSH into cumulative distribution function values, to quantify the central sensitivity of THs (60). Subsequently, the parameterized thyroid feedback quartile index (PTFQI) derived from TFQI, along with the thyroid stimulating hormone index (TSHI) and the thyroid stimulating hormone T4 resistance index (TT4RI), form the core set of indicators for evaluating central sensitivity (60). Previous studies have shown that reduced central sensitivity to THs (as indicated by increased TSHI, TFQI and TT4RI) is significantly associated with MetS, CVD, and CKD, as well as all-cause mortality and cardiovascular disease mortality in CKD patients. For per standard deviation (SD) increase in TFQI, the risk of diabetes and MetS increases by 13% and 16%, respectively (60). For per SD increase in PTFQI, the risk of all-cause mortality and CVD mortality increases by 18% and 31%, respectively (61).

The gender and age differences in the correlation between thyroid hormone sensitivity and CKM syndrome were investigated by Jiang et al. with 8391 euthyroid participants (62). Reduced thyroid hormone sensitivity (higher TFQI, TSHI and TT4RI), were significantly associated with an increased risk of stage 2 CKM in men, while were less so in women. This gender disparity was more pronounced among those over 50 years old (62, 63). Previous studies have confirmed gender disparity of TFQI in glucolipid metabolism: TFQI using FT3 independently predicted visceral fat obesity in male T2DM patients but not females (63). These variations may stem from distinct hormonal profiles, vascular compliance characteristics, and immune responses patterns. Estrogen appears to act as a safeguard for cardioprotective potency in female through inflammatory regulation and enhancement of lipid homeostasis. Further research is necessary to explore the gender-specific mechanism by which THs affect CKM syndrome, thereby providing new insights for clinical interventions.

### 5.3 Prospects of targeting thyroid hormone receptor in the treatment of CKM

Currently, the therapeutic options for CKM include sodium-glucose cotransporter 2 (SGLT2) inhibitors, glucagon-like peptide-1 receptor agonists (GLP-1 RA), and non-steroidal mineralocorticoid receptor antagonists (such as finasteride), among others (64–66). These drugs have demonstrated effectiveness in delaying disease progression and reducing cardiovascular risks in different patient populations. More efforts are still needed to develop drugs targeting new therapeutic targets in order to improve the prognosis of more patients.

Thyroid hormone receptor β (THRβ) is mainly distributed in the liver. THR-β agonists, as a newly discovered drug, have been proven to have the potential to regulate liver metabolism and overall metabolic risks (67). Beyond its well-established roles in glucose and lipid metabolism, the liver act as a crucial endocrine regulator across obesity, CKD, CVD, and diabetes mellitus (67). In metabolic-related steatohepatitis, THR-β agonists promote the metabolism and consumption of liver fat, and improve progressive liver fibrosis on the other hand. Resmetirom, a selective THR-β agonist, has achieved a landmark approval from the Food and Drug Administration specifically for the management of metabolic dysfunction-associated steatosis hepatis (68). Considering the hub function of liver in the cardiovascular-renal-metabolic pathway, the direct impact of THRβ on the CKM endpoint requires further exploration.

## 6 Conclusion

The intricate interplay between aging-related thyroid dysfunction and CKM syndrome underscores pivotal role of THs in metabolic homeostasis. While overt thyroid disorders warrant treatment in older adults, management of subclinical dysfunction, particularly hypothyroidism, requires nuanced risk stratification based on TSH levels. Critical knowledge gaps persist for cardiovascular benefits, including optimal intervention timing for subclinical hypothyroidism in the elderly, as well as the exploration of the efficacy of targeting thyroid receptors in CKM syndrome. Future research should focus on multiple mechanistic links and personalized therapeutic approaches to mitigate age-associated CKM syndrome.
